# Evaluating child helmet protection and testing standards: A study using PIPER child head models aged 1.5, 3, 6, and 18 years

**DOI:** 10.1371/journal.pone.0286827

**Published:** 2024-01-02

**Authors:** Xiaogai Li, Anna von Schantz, Madelen Fahlstedt, Peter Halldin

**Affiliations:** 1 Division of Neuronic Engineering, Department of Biomedical Engineering and Health Systems, KTH Royal Institute of Technology, Huddinge, Sweden; 2 Mips AB, Täby, Sweden; Al Mansour University College-Baghdad-Iraq, IRAQ

## Abstract

The anatomy of children’s heads is unique and distinct from adults, with smaller and softer skulls and unfused fontanels and sutures. Despite this, most current helmet testing standards for children use the same peak linear acceleration threshold as for adults. It is unclear whether this is reasonable and otherwise what thresholds should be. To answer these questions, helmet-protected head responses for different ages are needed which is however lacking today. In this study, we apply continuously scalable PIPER child head models of 1.5, 3, and 6 years old (YO), and an upgraded 18YO to study child helmet protection under extensive linear and oblique impacts. The results of this study reveal an age-dependence trend in both global kinematics and tissue response, with younger children experiencing higher levels of acceleration and velocity, as well as increased skull stress and brain strain. These findings indicate the need for better protection for younger children, suggesting that youth helmets should have a lower linear kinematic threshold, with a preliminary value of 150g for 1.5-year-old helmets. However, the results also show a different trend in rotational kinematics, indicating that the threshold of rotational velocity for a 1.5YO is similar to that for adults. The results also support the current use of small-sized adult headforms for testing child helmets before new child headforms are available.

## 1. Introduction

The head is a frequently injured body region in cyclists where severe head injuries can lead to cause of death in this population [1–4]. Children appear to be overrepresented and at greater risk than adults [4, 5]. Both epidemiological and biomechanical studies have demonstrated the efficacy of helmets in reducing the incidences and severity of head injuries [2, 6–10]. In some countries, children are legally required to wear helmets [1]. Helmets protect the brain by absorbing energy during impact and must meet certain standards to ensure proper protection.

Currently, there are several helmet testing standards used globally, including ASTM F1447 (America), EN1078 (European), AS/NZS 2063 (Australian/New Zeeland standards). There are also standards explicitly for child helmets (EN1080 and ASTM F1898) and standards that cover both adult and children helmets, e,g, CAN/CSAD1 13.2-M (Canada). All standards require the helmet to pass attenuation impact tests where the helmet/headform assembly is dropped against an anvil and linear acceleration is measured. The major differences among different testing standards are: Drop height (impact velocity) and the corresponding pass/fail threshold; impact anvil type (flat, hemispherical, or kerbstone anvil depending on which standard); free or guided fall. For instance, in EN1078/EN1080, the acceleration threshold is 250g for both a 1.5 m drop (5.42 m/s) on a flat anvil and a 1.06 m drop (4.57 m/s) on a kerbstone anvil and the fall is free. American standard ASTM F1447 has a higher threshold with higher impact velocities and the fall is guided, being 300 g for 2.0 m drop (6.2 m/s) on a flat anvil and 1.2 m drop (4.8 m/s) on hemispherical/curbstone anvils. Most standards use headforms confirming EN 960 with varying masses and sizes ranging from 3.1 kg (circumference 49.4 cm) to 6.1 kg (circumference 62.5 cm).

Child helmet testing standards, such as EN1080 and ASTMF1898, are specifically designed for child helmets, but they do not differ significantly from the general standards. EN1080 has additional specifications to prevent playground strangulations with breakaway buckles, while ASTMF1898 has an additional specification on headform. Both standards have the same specifications on attention tests and the same thresholding as their corresponding general standards EN1078 and ASTM F1447. The Canadian standard (CAN/CSAD1 13.2-M) covers both adult helmets and child helmets for children over and under five years old with varying thresholds depending on helmet size and headform, ranging from 200 g for the smallest helmet in a 1.5 m drop on a flat anvil to 250 g force for adult helmets. The Japanese testing standard (JIS T 8134) also has a different threshold for adult and child helmets. The standard requires that the peak acceleration not exceed 400 g force in flat anvil drops from 1.6 m for adult helmets, and 150 g force for 1.4 m drop for child helmets. Currently, child helmets are tested using small-sized adult headforms (e.g., 5^th^ percentile HIII). Working group 11 (WG11) of the European Standardization Head Protection committee (CEN/TC158) is developing new headforms with a more realistic moment of inertia (MoI) and coefficient of friction (CoF).

Compared with existing research on adult helmets, studies on child helmet protection are limited. Child helmets on the market for the youngest starting from 46-52/48-52 cm head circumference, suitable for children from 1.5 years. Children’s heads are smaller and softer than adults’ with softer skull bones in the younger [11] and are anatomically unique with unfused fontanel and sutures in the youngest. Age-dependence of child head injury under helmet protected head is unknown, which has important implications for child helmet design and testing standard development.

This study aims to address the knowledge gaps regarding child helmet protection and testing standards. To achieve this goal, we utilized PIPER finite element (FE) head models that include fontanel and suture for the youngest ages of 1.5 and 3 years old. Additionally, we upgraded an 18-year-old PIPER head to bridge children and adults. The study hypothesizes that: (1) the youngest age group will experience higher peak linear acceleration and greater skull stress levels due to their smaller head size and unique skull anatomy; (2) the current peak linear acceleration threshold of 250 g force may be conservative in terms of protecting children from skull fractures. To test these hypotheses, we investigated helmet-protected head responses for different ages under extensive linear and oblique impacts using two models of children’s helmets that are commercially available.

## 2. Method

### 2.1 FE head models

#### 2.1.1 Head models of 1.5, 3, 6, and 18 years old

**Fig 1:**
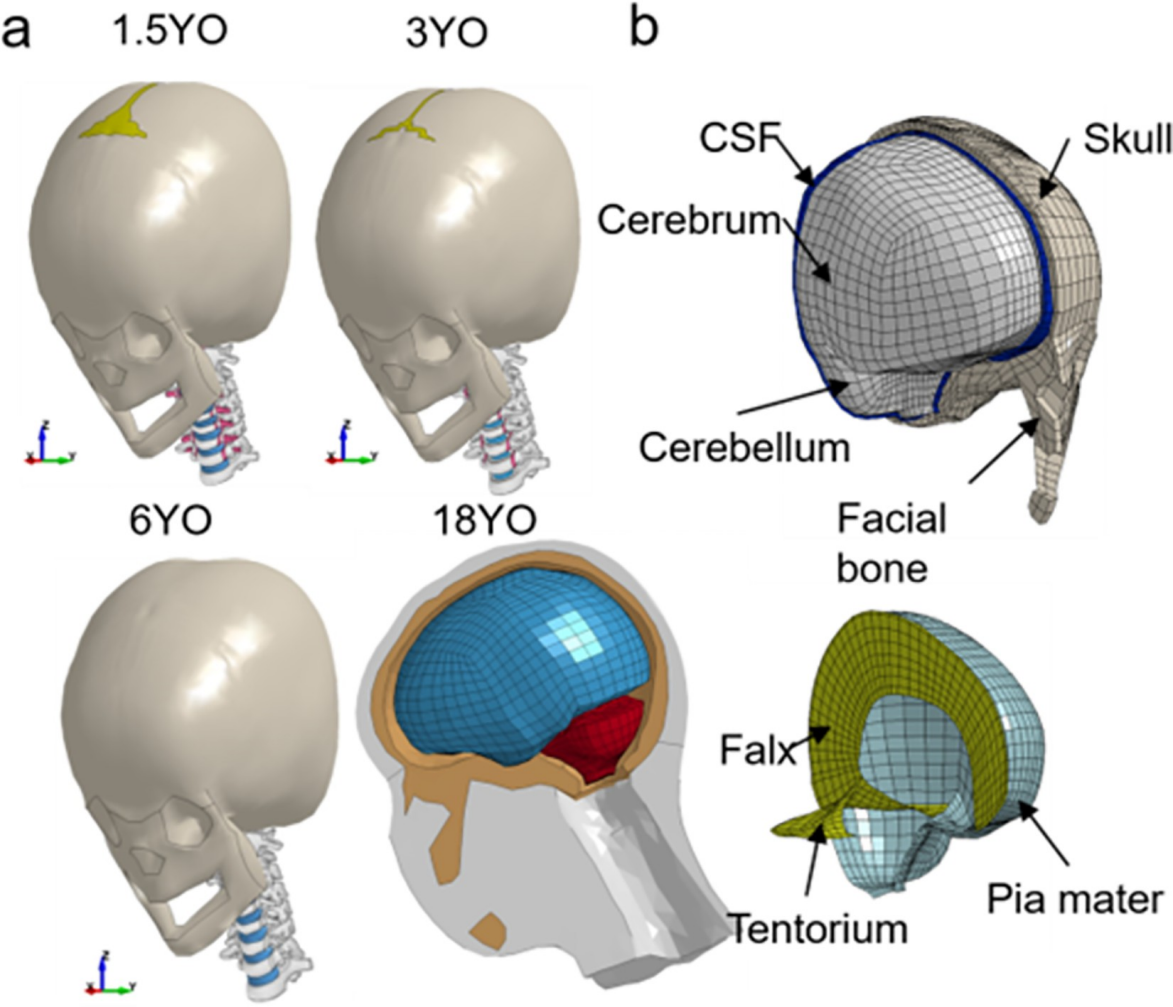
(a) FE head models of different ages, including the 1.5YO, 3YO, and 6YO showing continuous growth model accounting for the sutures in the head. Side view of the upgraded 18YO head model to highlight the corrected occipital shape after upgrading. (b) Isometric view of the head model illustrated with the baseline head model of a 6YO.

PIPER child head models are used in this study. A 6YO baseline PIPER child model was developed and released from the PIPER project [12]. Besides, a PIPER software is available to nonlinearly krige the baseline 6YO to different ages according to anthropometric data. The PIPER child model includes the whole body but only the head is used in this study. The head models of 1.5, 3, and 6YO presented earlier are reused here which has been improved than its released version with more geometrically correct tentorium, 2-layer skull, and nonlinear properties for soft tissues (details see [11]), which are re-used in this study. Besides these three ages, an 18YO head model is also included to bridge findings to adults. Though previous study [11] also presented an 18YO but is flat-headed and doesn’t represent a realistic head. Thus, in this study, we updated the geometry of the 18YO as described in the following section. **Table** 1 shows that these models have age-dependent material properties for the skull and suture, with the younger age group having softer bone and suture. However, other components of the models use the same material properties for all ages. The head models include the skull, brain, and facial bone (upper) and the brain is exposed to show the inner membranes of the falx, tentorium, and pia mater (lower) (**Fig 1**(B)). The performance of global impact kinematics has been assessed by comparing the model predictions of a 1.5YO and 6YO with those from cadaveric head tests of similar ages reported earlier [13]. Further details regarding the head model generation, material modeling, and validation are presented in an earlier study [11].

**Table 1:** Summary of material properties used in the head model.

Tissue	Material constants	*Density (kg/m* ^*3*^ *)*	Poisson’s ratio
Brain	*μ*_*1*_ = 53.8 *Pa*, *α*_*1*_ = 10.1, *μ*_*2*_ = -120.4 *Pa*, *α*_*2*_ = -12.9	1040.0	~0.5
CSF	*K* = 2.1 *GPa*	1000.0	0.5
Scalp connective tissue	*μ*_*1*_ = 1.30×10^4^ *Pa*, *α*_*1*_ = 24.2	1133.0	~0.5
Scalp adipose tissue	*μ*_*1*_ = 3.99×10^3^ *Pa*, *α*_*1*_ = 8.8	1133.0	~0.5
Dura mater, falx, tentorium	Hyperviscoelastic	1133.0	0.499
Pia mater	Hyperviscoelastic	1133.0	0.499
Skull	Age-dependent linear elastic* 6.30 GPa, 7.81 GPa, 9.32 GPa, 11.72 GPa (1.5YO, 3YO, 6YO, 18YO)	2000.0	0.22
Suture	Age-dependent linear elastic* 258.0 MPa, 533.0 MPa (1.5YO, 3YO)	1500.0	0.22

*Details see [11].

#### 2.1.2 Head model of the 18 YO and validation

**Fig 2:**
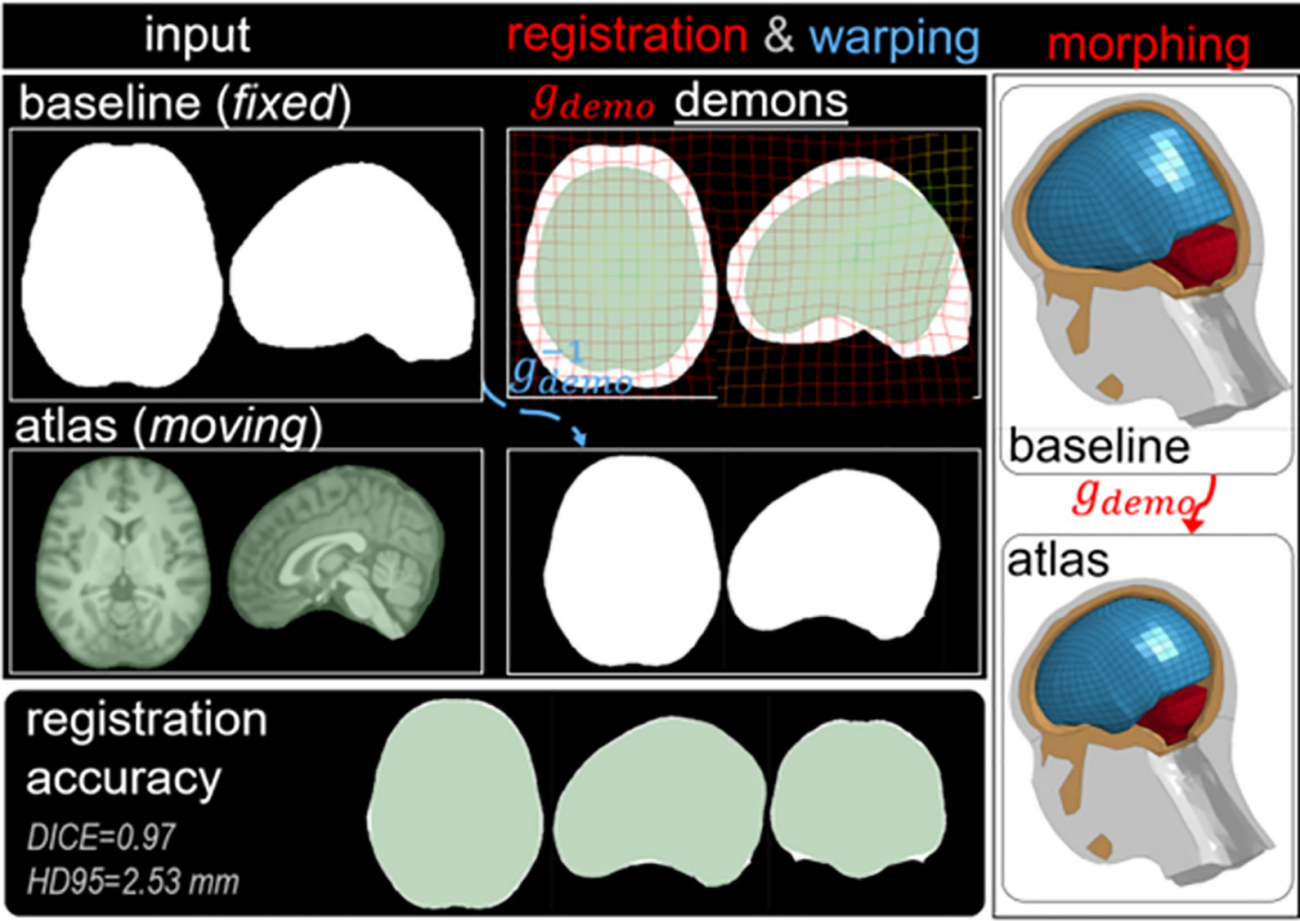
Mesh morphing pipeline to “correct” the PIPER software generated flat-headed 18YO, obtaining an upgraded 18YO head model.

The PIPER software though allows generating head models up to 18YO, which however has a very flat occipital lob due to the inherent limitation of the PIPER software that only implements a nonlinear kriging algorithm to 12 YO. Thus, in this study, we developed a mesh morphing pipeline to obtain an upgraded head model with geometry representative of an 18YO MRI atlas based on 107 subjects [14]. The pipeline allows morphing the PIPER software-generated flat-headed head according to the atlas using the Demons registration algorithm (**Fig 2**). Morphing accuracy is quantified by the *DICE* similarity coefficient and HD 95 distance [15, 16]. Briefly, the pipeline works as follows: First, the FE mesh of the cranial volume of the flat-headed head model is voxelized to a binary image. The MRI image of the 18YO atlas is also segmented for the cranial image. Second, Demons registration is performed with baseline as a *fixed* image and atlas cranial image as a *moving* image, from which a transformation *g_demons_* is obtained, which is then used to morph the baseline mesh resulting an upgraded mesh. The DICE value between the morphed mesh and the golden truth of the atlas is 0.97, and HD95 = 2.53mm, showing the upgraded head model represents the 18YO atlas well (**Fig 2**).

Brain tissue response of relative skull-brain motion predicted from the above upgraded 18YO head model is compared with measurements by Hardy et al. (2007) [17]. Three representative cases are chosen (sagittal impact C288-T3, coronal impact C380T-4, and lateral impact C380-T5). To identify the target positions and extract characteristic curves, the head model is scaled to match the experimental post-mortem head and the coordinates were expressed in relation to the head center. CORA scores are calculated to quantify the model performance on brain motion following the same procedure published earlier [15]. A summary of the biofidelity rating for skull-brain motion is listed in Table 2 and the CORA scores are comparable with previous models reported earlier [15].

**Table 2:** CORA score comparison between analysis for relative skull-brain motion.

Age	Test	CORA (upgraded head in this study)	CORA (Flat-headed before morphing [11])
18YO	C288-T3	0.507	0.527
	C380-T4	0.676	0.693
	C380-T5	0.605	0.585

Note: The CORA scores between the flat-headed and corrected head are close, which is not surprising. Because during validation, both heads have been scaled according to the global head size to the PMHS in Hardy study.

### 2.2 Helmet model development and validation

Two FE models of child bicycle helmets (*Helmet-A & B)* from Specialized® are developed based on a computer-aided design (CAD) model. The helmet models were selected based on their availability for purchase on the market and have been referred to as Helmet A and B to maintain anonymity. The *Helmet-A* suits for toddlers 1.5-4Y (46–51 cm), and *Helmet-B* suits for head circumferences between 50-55cm. Both helmet models included an outer shell and an expanded polystyrene (EPS) liner. The generated *Helmet-A* model consists of 260230 tetrahedral and 23050 shell elements, and the *Helmet-B* model consists of 215910 tetrahedral and 13626 shell elements. The outer shell was modeled with an elastic material with a thickness of 0.5 mm (MAT3), and the EPS liner is modeled with a foam material (MAT075) in LS Dyna [18]).

The helmet model is validated against experimental tests of dummy head drops. For this, experimental drop tests of *Helmet-B* were carried out at the Mips AB test laboratory. The helmet was placed on a 5th percentile Hybrid III headform and subjected to three oblique impacts (front, lateral, and pitched) and one linear impact (crown) as illustrated in **S1 File**. Two *Helmet-B* were used in the tests, with the first being subjected to three oblique impacts and the second subject to one linear impact. Linear acceleration, angular acceleration, and angular velocity of the headform were recorded and compared with that from the simulation. The data was filtered with an SAE 180 filter before analysis. CORA scores between the time-history curves of the linear acceleration, angular acceleration, and angular components are calculated and shown in Table 3. The average of all the impact locations is 0.76 for linear acceleration, 0.62 for angular acceleration, and 0.70 for angular velocity, suggesting a reasonable correlation between experiments and simulations and this validated the helmet model. Further details on helmet modeling and validation are provided in **Fig A1-A3 in S1 File.**

**Table 3:** CORA scores between experimental and simulation results for helmet validation drop test.

impacts	linear ace.	rot. accel	rot. velocity
Oblique front	0.83	0.53	0.62
Oblique lateral	0.84	0.50	0.80
Oblique pitched	0.71	0.82	0.68
Linear crown	0.65	-	-
**Average**	0.76	0.62	0.70

### 2.3 Helmet fitting

**Fig 3:**
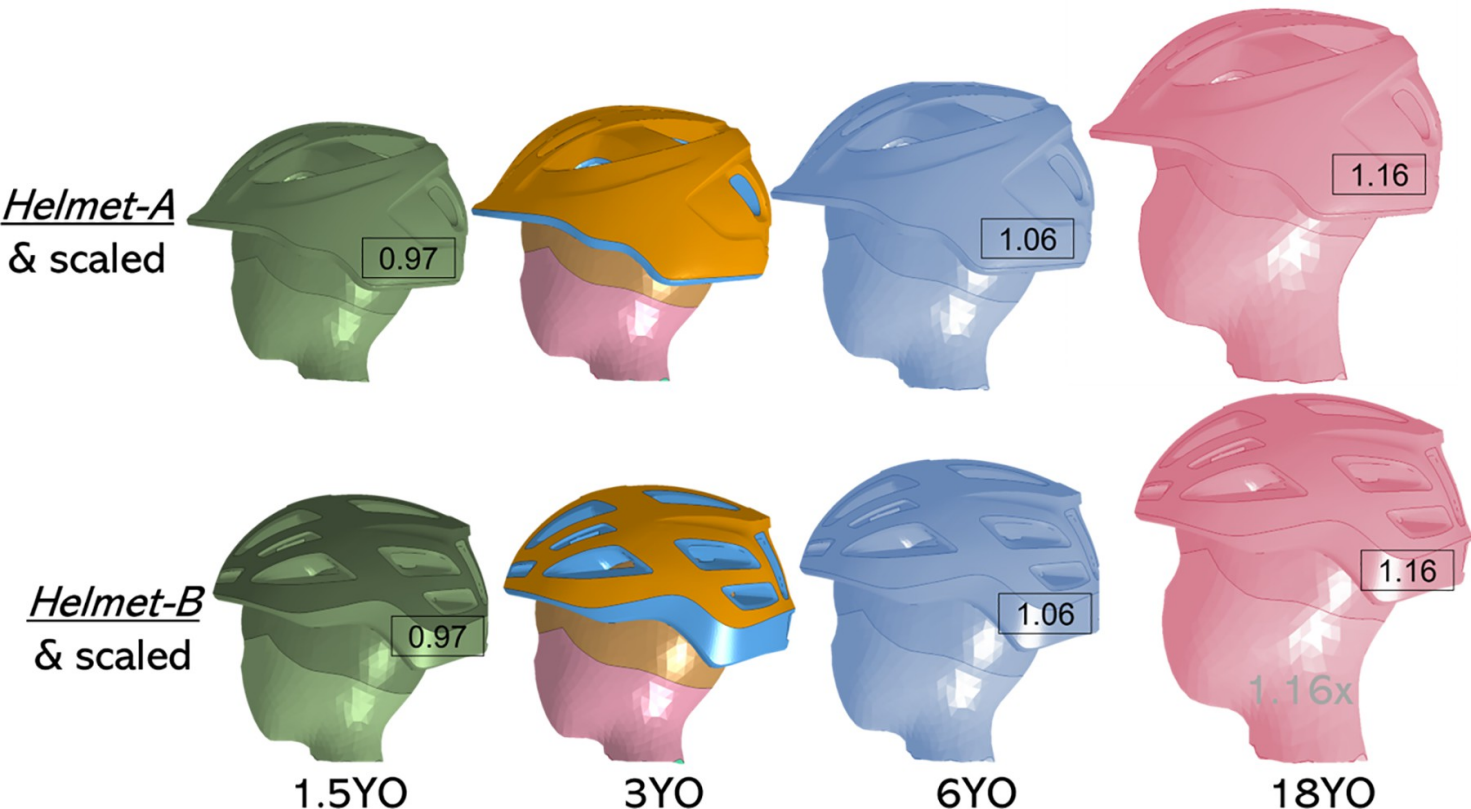
The baseline *Helmet-A* (upper row) and *B* (lower row) fit on the 3YO head and scaled helmets model are fitted to 1.5, 6, and 18YO with factors as indicated. The factors are calculated as head circumference between different ages divided by that of the 3YO (49.8, 51.2, 54.3, and 59.6 cm for the 1.5, 3, 6, and 18YO respectively).

*Helmet-A* and *B* models fit the head size of the 3YO model, while too large for 1.5YO, and too small for 6 and 18YO. Thus, the baseline *Helmet-A* and *B* are assembled to the 3YO head model. For other ages, to eliminate the potential influence of improper helmet fitting when studying the age-dependent head response, both helmets are first scaled globally before assembling into head models. The scale factor is calculated as the ratio of head circumference between different ages with the 3YO model as a baseline, which are 0.97, 1.06, and 1.16 respectively for the 1.5, 6, and 18YO. The same scaling factor is applied to both helmets, as they are of similar size despite different models. For all ages, the helmet is fitted to the head so that the front and top of the head are in contact with the helmet, representing a typical fitting when wearing a helmet (**Fig 3**).

### 2.4 Simulation matrix setup and evaluation of results

Each head and helmet assembly are subjected to in total of 15 impacts including 12 linear (5.4 m/s) and 3 oblique (6.2 m/s) impacts. Linear impacts consist of front, side, crown, and rear impacts at a flat anvil, kerbstone, and its alternative by rotating 90 degrees denoted as kerb.rot hereafter. Oblique impacts consist of front, lateral and pitch impact at a flat anvil. An illustration of the simulation setup is shown in **Fig A4 in S1 File**. Note that a different terminology, Xrot, Yrot and Zrot, has been used previously for oblique impacts [7] which correspond to oblique lateral, front and pitched in the current study.

All simulations were performed using LS-Dyna (version 971 revision 9.1) and post-processed with LS-PrePost version 4.3. The anvil is modeled as a rigid material. The coefficient of friction (COF) between the helmet EPS linear and the scalp is set to 0.3 according to the cadaver head experimental results of the scalp-liner COF reported earlier [19]. The COF between the helmet shell and anvil is set to 0.8.

Linear, angular accelerations and angular velocities are extracted from the accelerometer implemented at the center of gravity of the PIPER head model filtered by an SAE filter with a cut-off frequency of 180 Hz. 95th percentile maximum 1st principal Green-Lagrangian (G-L) strain (referred to as 95^th^ brain strain) is extracted in the brain following previous studies to avoid potential numerical issues [11]. Maximum von Mises (v-M) stress in the skull bone during the entire impact is also extracted.

## 3. Results

### 3.1 Linear impacts: Linear acceleration and skull stress are age-dependent

**Fig 4:**
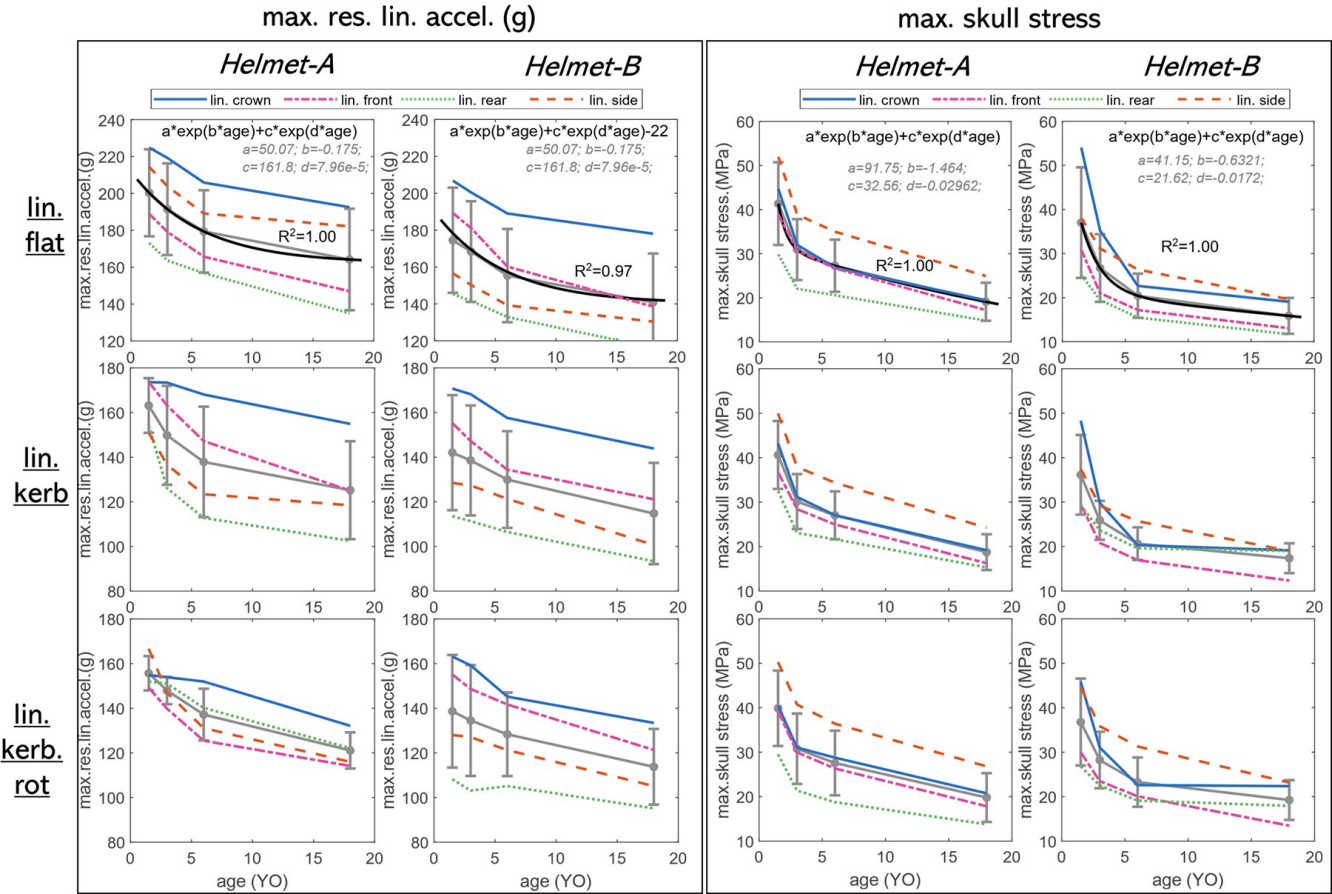
Age dependence of maximum res.lin.accel (left column) and max. skull stress (right column) for all linear impacts (row 1: flat anvil; row 2: kerbstone anvil; row 3: kerbstone-rotated anvil). Error bars are plotted together with the average values between different impacts plotted as gray lines. The back line in the first row is the fitted curve with coefficients indicated.

For drop impacts at the fixed linear anvil (flat, kerbstone, kerbstone rotated), there is a clear trend that the younger age has higher peak linear acceleration and skull stress, with the youngest being the most vulnerable. The maximum resultant linear acceleration (max.res.lin.acc) in terms of peak linear acceleration (hereafter abbreviated as peak *g*) decreases with age for all linear impacts (**Fig 4**, left column). The maximum skull stress also decreases with age (**Fig 4**, right column). This trend holds for both *Helmet-A* and *B* and all evaluated linear impacts. In particular, the average peak linear acceleration and maximum skull stress at all four impact locations (crown, front, rear, side) show an exponentially decreasing trend, with skull stress having a deeper decrease, as shown for impact at the flat anvil (**Fig 4** row 1) with fitted curve indicated. Note both helmets show peak linear acceleration decreases with age, though *Helmet-B* on average appears to have better protection with a lower peak linear acceleration for all impact locations, the fitted curve of average being 22g lower for flat anvil (**Fig 4** row 1). The decreasing trend holds for the impact at kerbstone and kerbstone-rotated, and the orientation of kerbstone influences the response though not considered as significant (**Fig 4** rows 2&3).

### 3.2 Oblique impacts: Rotational kinematics and max. brain strain

**Fig 5:**
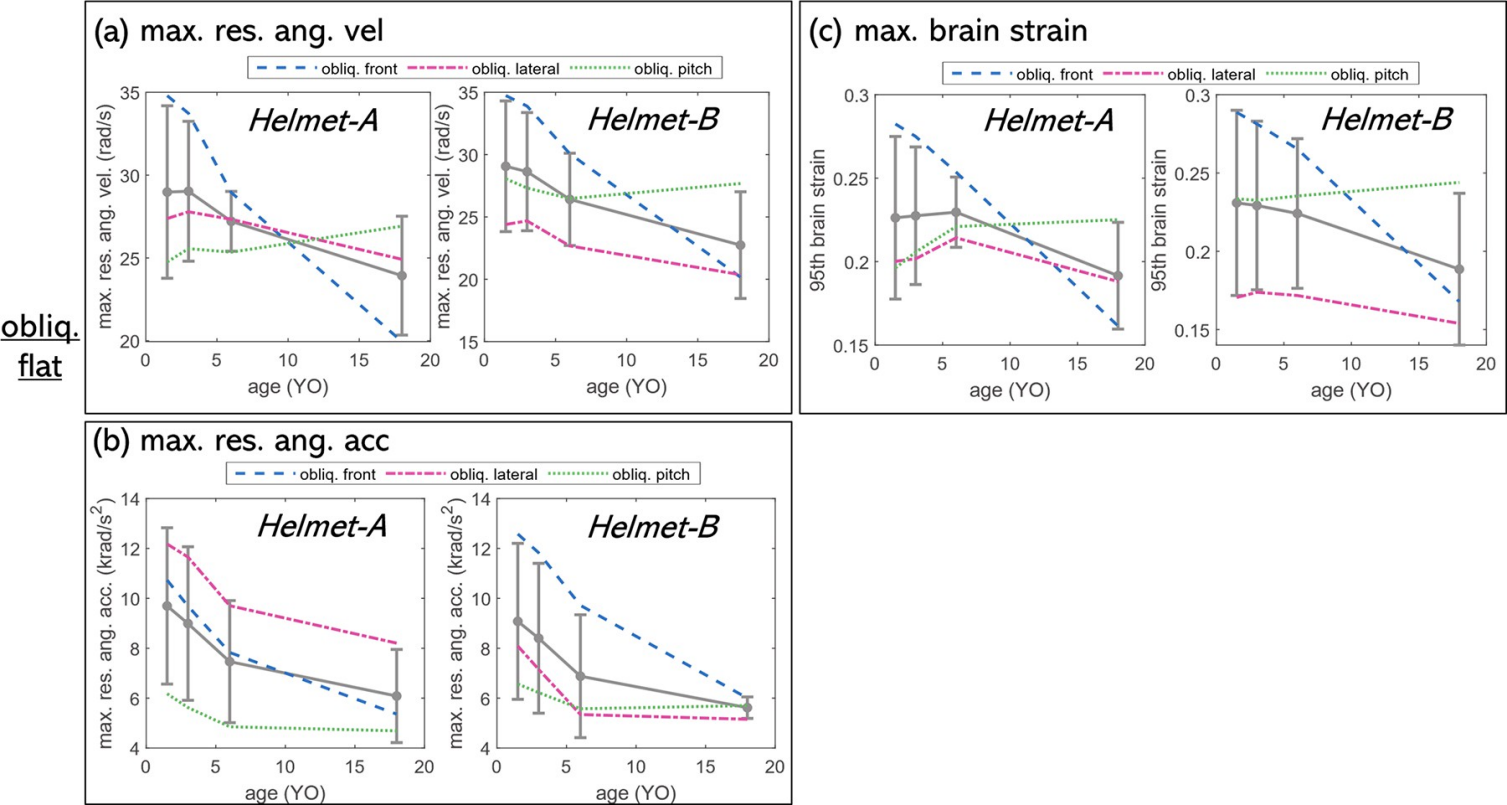
Age dependence of (a) max.res.ang.vel & (b) max.res.ang.acc (b) and (c) maximum brain strain.

Angular acceleration decreases with age (**Fig 5**B), but the age trend for angular velocity (**Fig 5**A) and brain strain (**Fig 5**C) is not monotonic and depends on impact locations for both helmets. For example, both velocity and brain strain show a clear decreasing trend with age only for oblique front impact (**Fig 5**A dashed blue curve), while for oblique lateral impact, both velocity and strain increase and reach the maximum for 3YO and then decrease with age. Further, for oblique pitched impact, both velocity and brain strain increase with age, with the largest head 18YO largest brain strain (**Fig 5**C). The maximum resultant angular velocity max.res.ang.vel correlates better with brain strain compared to maximum angular acceleration (max.ang.acc.) for most cases (**Fig 5**A and **5**C). Note that age influences on max.ang.vel (**Fig 5**A) and brain strain (**Fig 5**C) are the largest for front impact compared with lateral and pitched impact. This could be due to the inherent fitting of the helmet with a larger gap between front-back (corresponding to front impact) than the side (corresponding to lateral and pitch) since the same impact points were assured for different ages. Helmet shape differences at impact sides of front, lateral, and pitched could also cause a different age influence.

### 3.3 Skull stress and brain strain analysis

**Fig 6:**
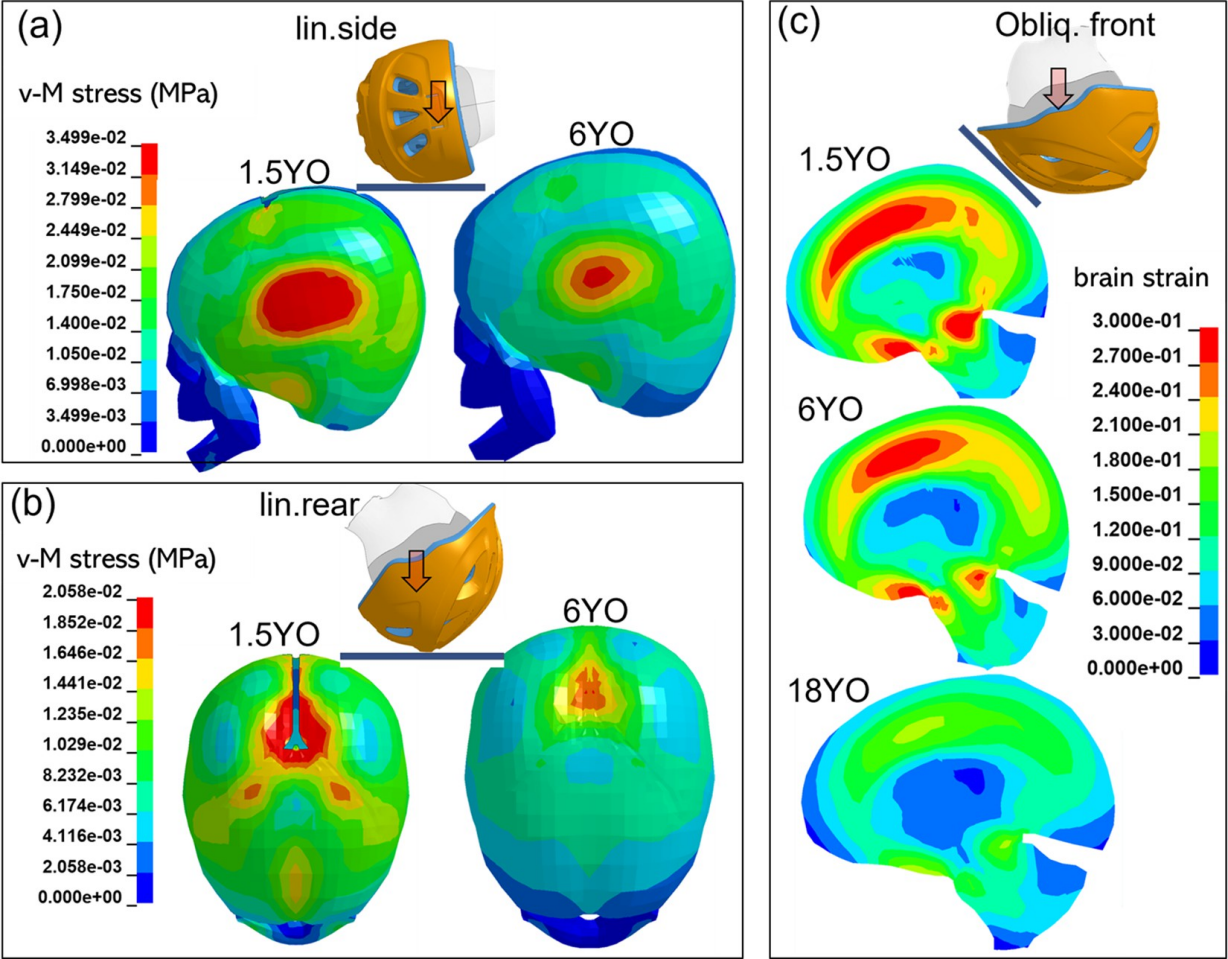
Age-dependence maximum v-M stress in the skull (a,b) and of brain strain (c) with *Helmet-A*. A sagittal plane of brain strain (1.5, 6, and 18YO oblique front) and skull stress (1.5 and 6YO linear side and linear rear) captured when peak value occurs (illustration with *Helmet-A*).

To further understand the age-dependent trend, three impacts are selected for analysis: two linear impacts for analyzing skull stress and one oblique impact for analyzing brain strain, as linear impacts are more relevant to skull stress, while the oblique impact is more relevant to brain strain. Results from *Helmet-A* are presented in **Fig 6**, and results from *Helmet-B* are found in **S2 File**. Specifically, the findings from both helmets indicate that younger individuals have higher skull stress and brain strain from helmets. Skull stress contours for the 1.5 and 6YO are extracted for linear side and linear rear impacts showing for both impact locations and both ages, the highest skull stress is located close to the initial impact point (**Fig 6**A and **6**B). The largest stress in the youngest seems to be attributed to multiple factors. First is the presence of suture, e.g., for impact at the rear head, the stress peaks near the suture/fontanel edge (**Fig 6**A). Note also that the 1.5YO has the highest peak stress which is known to correlate with skull fracture. Similarly, the brain strain contours are further analyzed for oblique frontal impact for the 1.5, 6, and 18YO, showing the largest strain in the smallest head and for different ages, the brain areas with high strain are located at the same location just that the levels decrease with age (**Fig 6**C), the same trend with rotational velocity (**Fig 5**C blue dashed line).

### 3.4 Implications for child helmet testing standard

**Fig 7:**
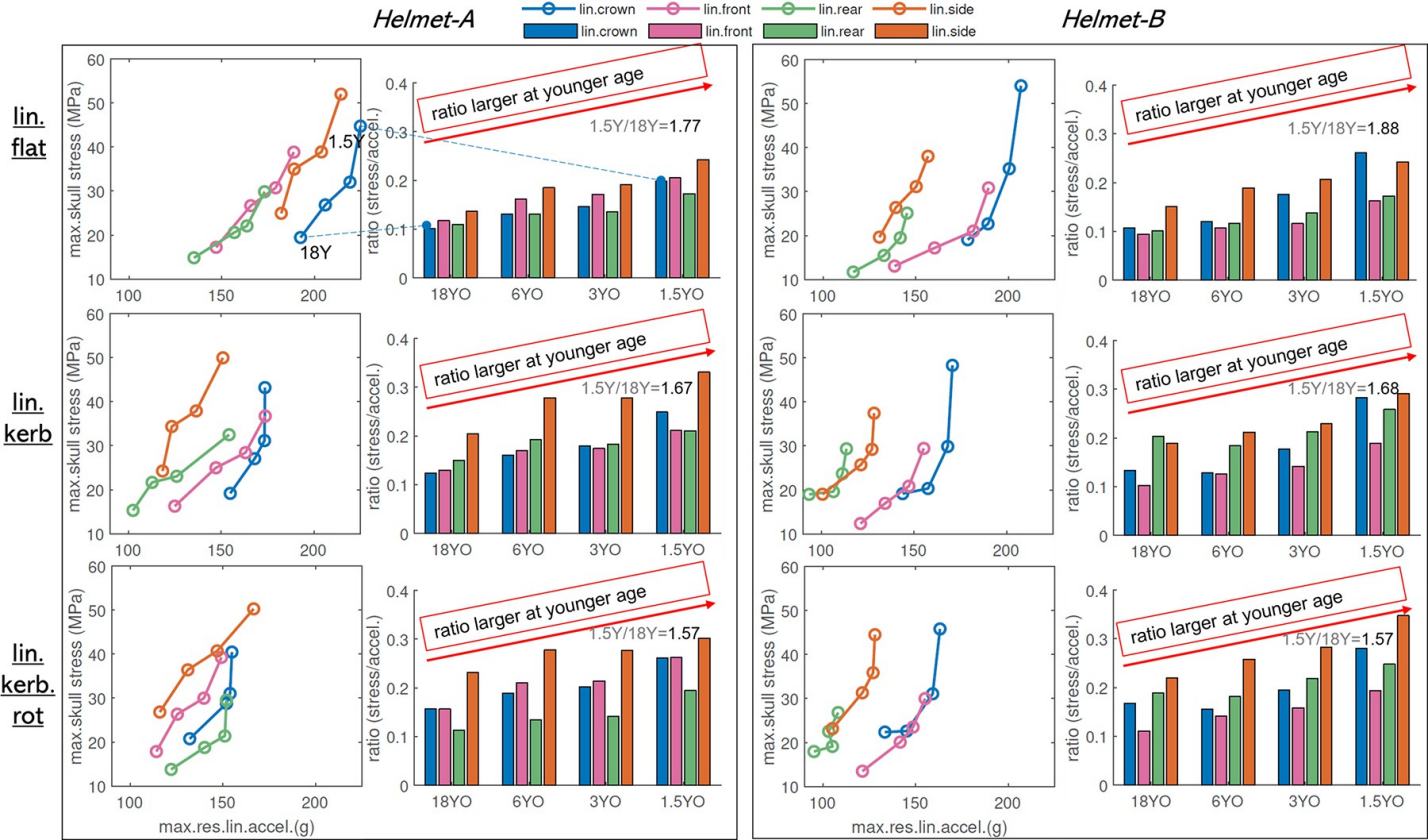
Correlation between max.res.lin.accel and skull stress for *Helmet-A* with each circle representing one age (column 1) at one impact location; Bar plot of *ratio* calculated as max.res.lin.accel (g)/max.skull stress (MPa) for each age and all 4 linear impact locations including crown, front, rear, and side (column 2). Each circle on column 1 corresponds to one bar on the right bar plot, e.g. as indicated for the 1.5YO and 18YO front impact (blue color). The *factor* indicated in each bar plot is defined as *mean ratio* of the 4 impacts for the 1.5YO divided by the corresponding value for the 18YO. For example, for a linear flat anvil (**row 1**), the mean ratio for the 1.5YO is 0.206 and the mean ratio for the 18YO is 0.116, resulting in a value of 1.77 representing the difference between 1.5YO and 18YO. Similarly, the *factor* for linear kerbstone and kerbstone rotated are calculated as 1.67 and 1.57 respectively. The same analysis is done for *Helmet-B* resulting in 1.66, 1.66, and 1.57.

To assess whether the same threshold of 250g would be reasonable for younger helmets, the *ratio* between maximum skull stress and acceleration (defined as *ratio* = max.res.lin.accel (g)/max.skull stress (MPa)) is plotted for all the four linear impacts (**Fig 7**). The *ratio* is shown to have an age dependence and in general with larger values at younger ages. The mean ratio between the 4 impact locations for each age is also calculated showing mono decreasing trend with age, the 18YO has the lowest *ratio*, and 1.5YO with the largest, meaning under the same peak linear acceleration the 1.5YO has higher skull stress. Based on *Helmet-A*, the *factors* are calculated as 1.77, 1.67, 1.57 respectively for the linear flat, linear kerbstone and linear kerbstone rotated respectively, and the corresponding factors from *Helmet-B* are 1.66, 1.66, and 1.57. Thus, a factor of 1.68 is calculated as the mean of all the above six values, meaning if assuming the same skull failure stress for all ages, the peak linear acceleration for the youngest 1.5YO should be adjusted by a factor of 1.68, resulting in a proposed threshold of 250/1.68 close to150g.

**Fig 8:**
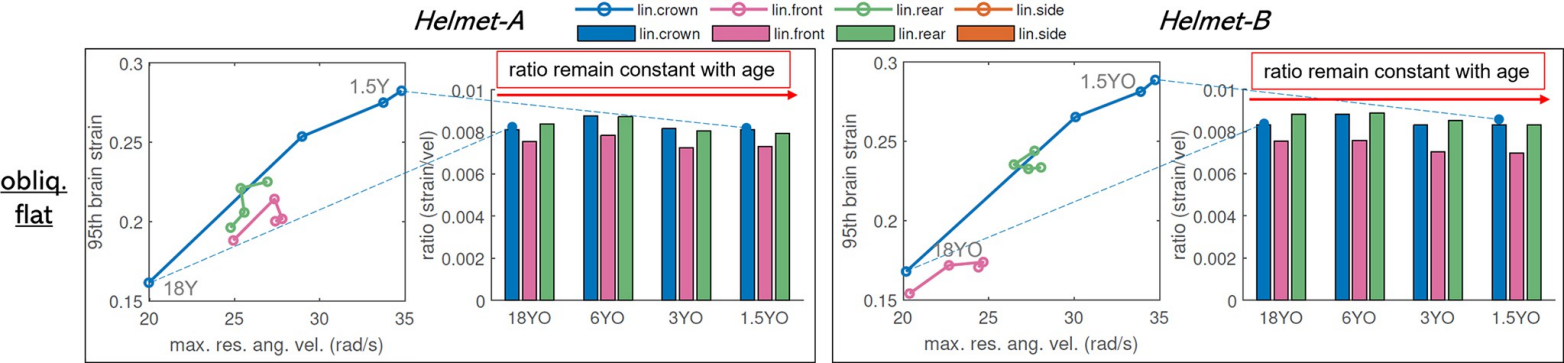
Correlation between max.res.ang.vel and brain strain. The relatively constant relation between brain strain and rotational velocity suggests a similar pass/fail criteria of rotational velocity for child helmets as adults.

The correlation between rotational velocity and brain strain is analyzed similarly as above for the three oblique impacts (**Fig 8**). Interestingly, for both helmets, the ratio (defined as *ratio* = max.res.ang.vel(rad/s)/95 brain strain) in general keeps at a constant value among all ages. This result suggests that the kinematic metric based on the rotational velocity could be the same for child helmets as for adults.

## 4. Discussion

### 4.1 Age-dependence of brain strain for helmeted protected head

FE head models have been increasingly used in evaluating helmet protection and ranking helmets during the last decades (e.g., [7, 20]) mostly on adult helmets. Comparatively studies on child helmets are limited. The PIPER child model is a continuously scalable HBM that scales a baseline of 6 years to varying ages [12]. The model responses have been compared to experimental studies for all body regions, showing a good performance including drop and compression tests for the head; bending and tensile tests for the cervical spine; pendulum and belt interaction tests for the trunk; bending tests for the lower extremities and full body sled tests for the mobility of the spine; side impacts for shoulder and pelvis. The model has been used in a variety of applications such as vehicle accident reconstructions [21] and playground head injuries in children [11, 22].

In this study, the largest brain strain occurs in the youngest 1.5YO in all evaluated oblique impacts and for both *Helmet-A* and *B* (**Fig 5**C). This may appear contradictory to previous studies, which that a larger brain tends to increase brain strains under the same loading [15, 23–25]. For instance, globally scaling an adult FE head model to six heads of varying dimensions showed brain response increased almost monotonically from the smallest to the largest head under a linear acceleration [23]. Similarly, nonlinearly morphed adult head models show the same trend though not necessarily monotonic due to individual brain structure differences [15].

The difference between this current study and previous studies lies in the loading applied, apart from the use of helmets. In previous studies, the loading was applied as inertial loading i.e., the same accelerations to the C.G. center of the head, whereas this study evaluates helmeted head impacts by applying the same drop height/initial velocity impacted at an oblique anvil. The smaller head with lower mass rotates more than a larger head, as simulation results show (**Fig 5**A & **5**B), resulting in larger rotational kinematics and therefore a larger brain strain. Other factors such as the youngest age with unfused fontanel/suture and a softer skull bone may also contribute to the higher strain in the youngest, which could be further clarified with a parametric study with fontanel/suture fused. However, these factors are considered insignificant, especially since the head is wearing a helmet, since the softer foam in the helmet appears to *shield* the differences in skull bone properties in head models (see **Sec 4.3**).

### 4.2 Age-dependence of skull stress for helmeted protected head

In all the evaluated linear impacts and for both *Helmet-A* and *B*, the youngest 1.5YO has the largest peak linear acceleration even with the softest skull bone. The age-dependent trend is like previous findings for non-helmeted head impact onto flat playground impact [11, 22]. A plausible explanation could be that the youngest has the smallest size. The skull stress follows a similar age-dependence trend as peak g. Larger skull stress in younger age is expected to be partially caused by larger peak g. The presence of suture in the youngest age of 1.5, and 3YO further increases the peak stress in the youngest (see Sec 3.3 Skull stress and brain strain analysis for further analysis), causing an even deeper decreasing trend in the youngest than peak g. It’s interesting to note that for rear impact, the stress peaks far from the suture also lead to larger stress in smaller heads (**Fig 6**B). This could be due to the smaller head size (smallest head with the largest acceleration which then often related to skull stress) despite a softer bone (6.30 GPa for 1.5YO and 9.32 GPa for 6YO).

Though a clear trend is found for age-dependence on both max.res.lin.acc and skull stress, the influence of impact locations is more complicated, that an impact location with the largest max.res.lin.acc does not necessarily lead to the largest v-M stress among locations. For instance, for *Helmet-A* linear impact, max.res.lin.acc is the largest for front impact (**Fig 4** row 1 left box), while the largest skull stress is found in a linear side impact (**Fig 4** row 1 right box). The reason is multifactor, which could be due to helmet thickness, and curvature of skull bone at impact sites between front and side impact. Particularly, side impact seems to have the highest skull stress for most cases, except for some of the youngest where the crown has the highest skull stress with *Helmet-B* (**Fig 4** row 1–3 right box).

### 4.3 Implications of child helmet testing standard threshold

Most current helmet testing standards adopt the same peak linear acceleration for child helmets as adults. However, the current results show that the youngest 1.5YO has the largest peak linear acceleration and skull stress, indicating the youngest skull needs better helmet protection and advocates a lower threshold than the adult helmet. Linear acceleration is associated with the risk of skull fracture and a previous study estimated a peak acceleration of 250g causing a 4% risk of skull fracture [26]. According to the 12 linear impacts at a flat anvil, kerbstone, and kerbstone rotated (each with four impact locations including front, side, crown, and rear), a threshold of 150g is proposed for the youngest helmet of 1.5YO based on a scale factor calculated between 1.5YO and 18YO (**Sec 3.4**). The proposed 150g is based on assumption that different ages have the same failure skull stress. Note the threshold is a preliminary value as it’s based on maximum skull stress which may have numerical instabilities at suture/bone edge with current mesh density, and percentile stresses could be evaluated similarly as brain strain uses 95^th^ values. For oblique impacts evaluated, the ratio between brain strain and rotational kinematics keeps constant across ages. Thus, the threshold of the kinematic metric based on the rotational velocity could be the same for children’s helmets as for adults.

### 4.4 Implications on child headform for helmet testing due to "helmet shielding” effect

When testing child helmets, 5^th^ percentile female headform has been used, e.g., as used in a consumer test [27], and the same as in the current study. The question is whether or not a unique headform for the youngest is needed considering its softer skull bone. An internal test shows that by comparing the baseline 1.5YO head model with unfused fontanel/suture and softer skull bone (Young’s modulus E = 6.30 GPa) to a rigid skull, the resultant difference in brain strain is minimal, e.g., for a linear crown impact. However, when both heads are without a helmet, the model with rigid skull bone could have more than double the peak linear acceleration than the baseline 1.5YO with a softer skull. This indicates that the helmet foam has shielded the difference between a softer and rigid skull bone, which we define as the “helmet shielding” effect in this study. Thus, due to “helmet shielding”, it appears that no unique child headform is needed as long as the size is fitted to the child helmet, and these results support the use of 5^th^ percentile headform for child helmet testing before new child headforms are available which is one of the tasks for WG11.

### 4.5 The upgraded 18YO model

To bridge child helmet and adult helmet evaluation, a comparable adult head model is needed with similar mesh density and morphology. An 18YO head model though possible to be output from the PIPER software, nevertheless has an unrealistically flat occipital lobe and larger head size, and the incorrect geometry of the 18YO head model may jeopardize model performance. In this study, we upgraded the flat-headed 18YO PIPER head model directly from the PIPER tool by an extra morphing step, and the resultant 18YO model now has a shape and size corresponding to a representative 18YO atlas. The model is re-validated against Hardy data [17] providing a good basis for bridging head responses between child and adult head models.

### 4.6 Implications on child helmet design

This study includes two child helmets, *Helmet-A* and *B*. Note both helmets show peak linear acceleration decreases with age, though *Helmet-B* on average appears to perform better in lowering peak linear acceleration and skull stress for linear impacts, e.g. 22g lower than *Helmet-A* (**Fig 4** row 1), further visualized in the stress contour figures (Fig 6). Further, for linear flat anvils, *Helmet-A* leads to the second highest max.res.lin.acc for side impact whereas *Helmet-B* has the second highest for front impact (**Fig 4** row 1). A different trend is seen for lin.kerb and lin.kerb.rotated for different impact locations (**Fig 4** rows 2&3). Both helmets have similar performance in reducing rotational kinematics and brain strain overall (Fig 5), though for specific impacts, especially interesting to note that *Helmet-A* outperforms *Helmet-B* in reducing brain strain. Learning the difference between two designs with child head models would offer an opportunity for improved child helmet design.

### 4.7 Limitations and future work

The scaling of helmets from the baseline of *Helmet-A* and *B* is to ensure the same fitting between the helmet and the head thus eliminating the potential compounding factors due to the fitting. This leads to a focused study on age dependence only. Despite the scaled helmet may not exist, a parametric study could be performed with the closest real helmet size to the scaled version, also note only the baseline helmet fitted to the 3YO has been validated. We attempt to propose a new threshold of 150g as a pass and failure threshold for the youngest helmet. Note the threshold is to be seen as a preliminary as it is based on the number of impact locations and helmets evaluated. Though we performed an extensive impact matrix (linear impacts with flat, kerbstone, kerbstone rotated anvil) and oblique impact (front, oblique, and pitched) with two helmet child helmets, further work needs to be done including more comprehensive impacts and child helmet models. Nevertheless, this current work serves as a first initiative towards a refined pass/fail threshold for child helmets.

The present study did not consider gender differences between boys and girls, as the PIPER head models used were generated directly from the PIPER software, which does not differentiate between the two. Additionally, the head models used in this study are nearly symmetrical which is based on anthropometric data that corresponds to a specific age group. However, children’s heads tend to be asymmetrical, which can affect impact responses. Future research could examine the impact of head shape differences in boys and girls, also asymmetry by using subject-specific models. Furthermore, the PIPER child head model was utilized in this study due to its open-source nature and the ability to continuously scale it through the accompanying PIPER software. The head model has also been previously validated. Future research could explore the same topic using other child head models. Two models of children’s helmets (*Helmet-A* and *Helmet-B*) were selected from the market. A validation drop test was performed only for *Helmet-B*, it would have been desirable to conduct a similar test for *Helmet-A* as well to ensure the performance of this helmet model.

## 5. Conclusion

This study provides the first computational evidence for age-dependence head response in helmeted head impacts in children and has implications for child helmet design and testing standards. The results show global kinematics and local tissue response are age-dependence based with the youngest age most venerable with the highest skull stress and brain strain. The results from the current study evidence a lower linear pass/failure threshold for the youngest helmet with a preliminary value of 150g, while the same rotational velocity threshold applies for children same as adults. Further, the results support the current use of a small-sized adult headform for testing child helmets before new child headform are available.

## Supporting information

S1 File Helmet modeling, validation and simulation setup illustration. (DOCX)

S2 File Skull stress and brain strain from Helmet-B. (PDF)
