# Supplementary material for: Evaluating child helmet protection and testing standards: A study using PIPER child head models aged 1.5, 3, 6, and 18 years
Source: PLoS One. 2024 Jan 2;19(1):e0286827. doi: 10.1371/journal.pone.0286827 (PMC10760764; doi:10.1371/journal.pone.0286827)
Supplement: S1 File — (DOCX) [file pone.0286827.s001.docx]

Supplementary Information: S1 File


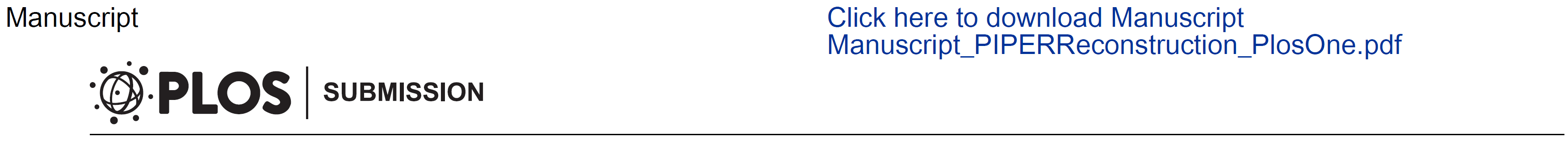


**Evaluating Child Helmet Protection and Testing Standards: A Study Using PIPER Child Head Models Aged 1.5, 3, 6, and 18 Years**

**Xiaogai Li^1^*, Anna von Schantz^2^, Madelen Fahlstedt^2^, Peter Halldin^1,2^**

^1^Division of Neuronic Engineering, Department of Biomedical Engineering and Health Systems, KTH Royal Institute of Technology, Huddinge, Sweden

^2^Mips AB, Täby, Sweden

*Correspondence: xiaogai@kth.se

# S1 File: Helmet modeling, validation and simulation setup illustration

The two developed FE models of child bicycle helmets (*Helmet-A & B)* from Specialized® is illustrated in **Fig.A1**. The material properties of helmet parts are presented in **Table A1**. The EPS liner is modeled with a foam material, namely MAT075 in LS Dyna (Hallquist, 2016). incorporating uniaxial and hydrostatic stress-strain curves shown in **Fig. A2**. Both helmet models used the same foam material property. The rationale for using this material model for helmeted head impacts is explained in detail earlier (Meng et al., 2019). Drop tests with helmet placed on a 5th percentile Hybrid III headform (**Fig.A3**) subjected to three oblique impacts (front, lateral, and pitched) and one linear impact (crown). Finally, the simulation


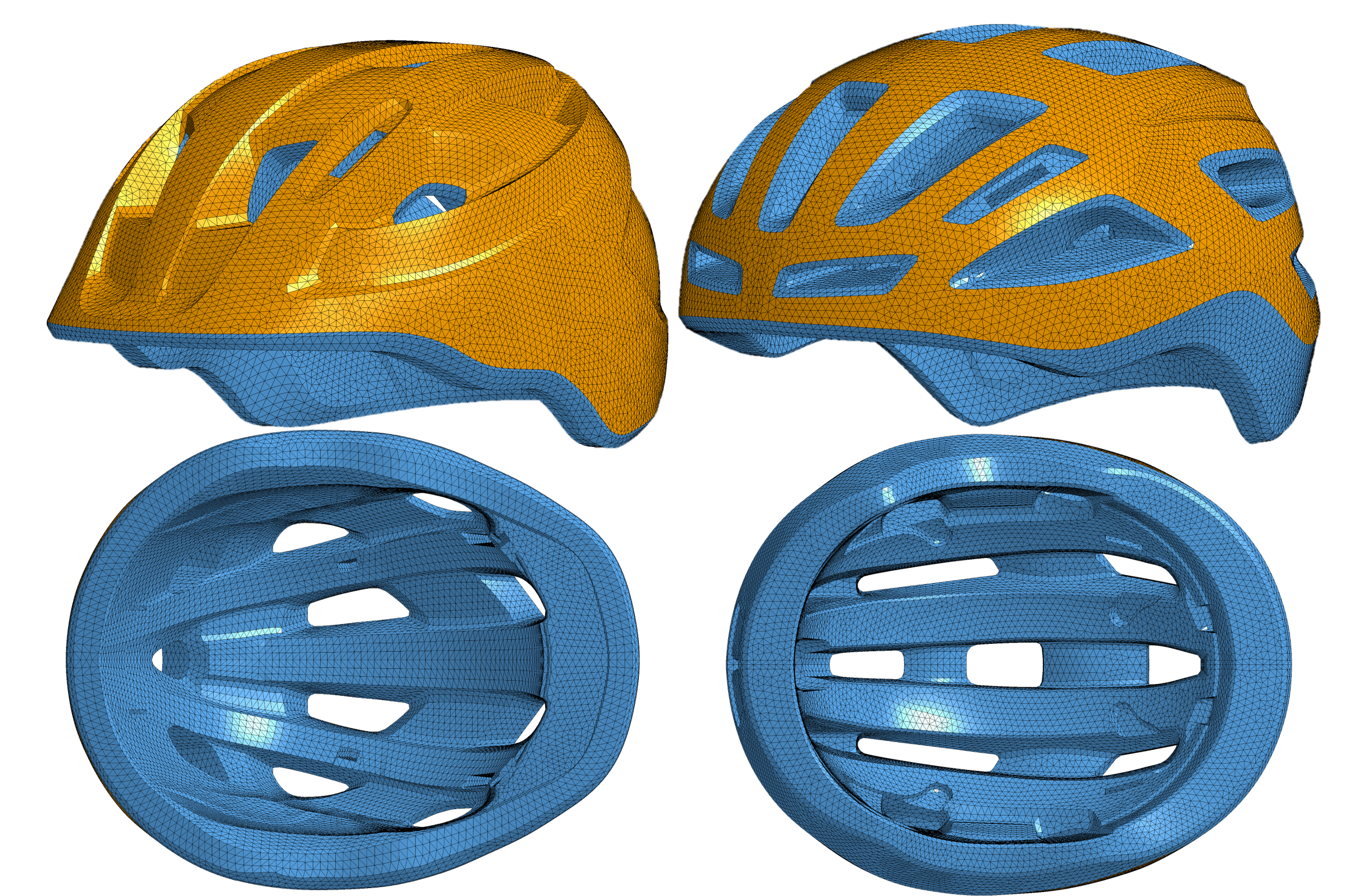


**Fig. A1** FE models of *Helmet-A* (on the left) and *Helmet-B* (on the right) showing the different designs. The outer shell is depicted in yellow, while the EPS is shown in blue.

**Table A1** Material properties of the helmet component.

|  | Density  (kg/m3) | Young’s modulus (MPa) | Poisson’s ratio | Yield stress  (MPa) | Tangent  modulus |
| --- | --- | --- | --- | --- | --- |
| EPS Liner | 75 | 41 |  |  |  |
| Outer shell | 1500 | 12e3 | 0.22 | 50 | 1 |


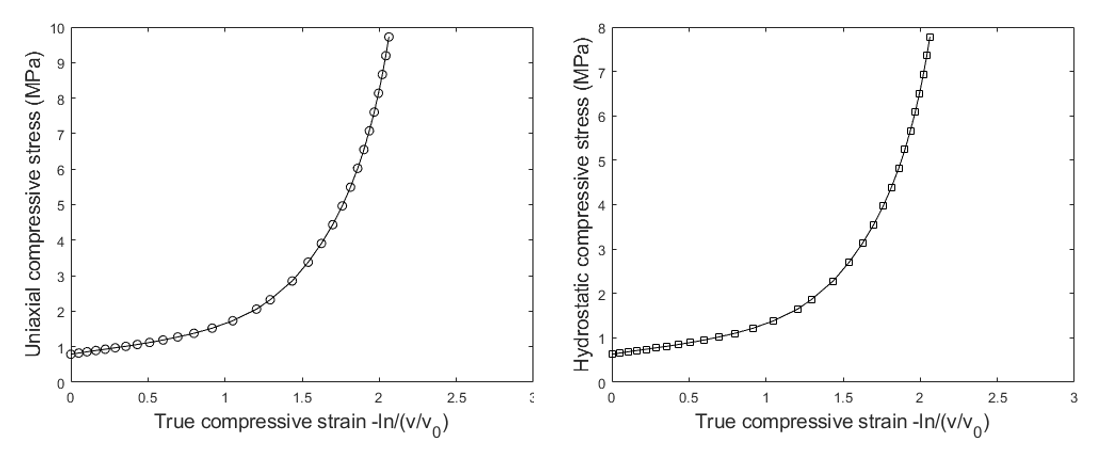


**Fig. A2** Uniaxial and hydrostatic stress-strain curves of EPS (75 kg/m^3^).


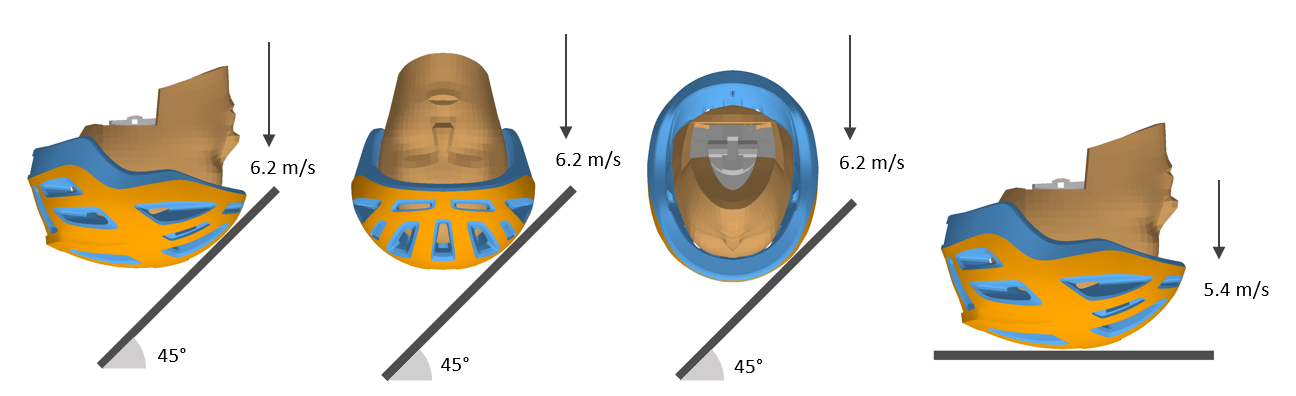


**Fig. A3** Setup of the helmet tests (from the left to the right oblique front, oblique lateral, oblique pitched, and linear crown) for helmet validation illustrated with numerical reconstructions of the physical dummy drop tests.


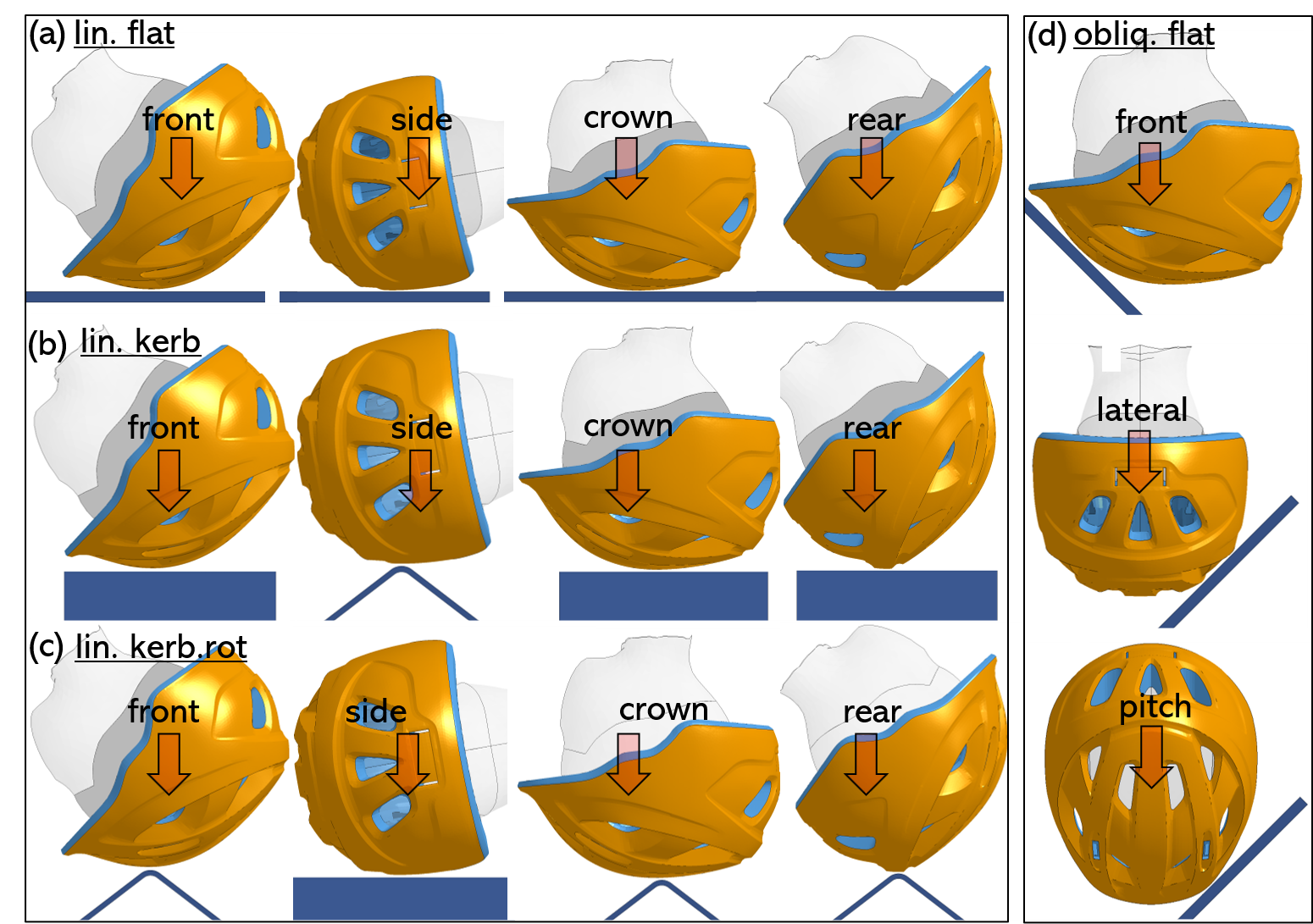


**Fig. A4** Each helmeted head model is subjected to 15 impacts including (a) linear impact on a flat anvil, linear impact on kerbstone (b), kerbstone rotated (c), and (d) oblique impacts on a flat anvil. Illustration with the 3YO head model wearing *Helmet-A*.

# References in S1

[1] J.O. Hallquist, LS-DYNA theory manual, Livermore software Technology corporation 3 (2006) 25-31.

[2] S. Meng, A. Cernicchi, S. Kleiven, P. Halldin, The biomechanical differences of shock absorption test methods in the US and European helmet standards, Int. J. Crashworthiness 24(4) (2019) 399-412.
