# Supplementary material for: Evaluating child helmet protection and testing standards: A study using PIPER child head models aged 1.5, 3, 6, and 18 years
Source: PLoS One. 2024 Jan 2;19(1):e0286827. doi: 10.1371/journal.pone.0286827 (PMC10760764; doi:10.1371/journal.pone.0286827)
Supplement: S2 File — (PDF) [file pone.0286827.s002.pdf]

Supplementary Information

PLOS | SUBMISSION

# Evaluating Child Helmet Protection and Testing Standards: A Study Using PIPER Child Head Models Aged 1.5, 3, 6, and 18 Years

**Xiaogai Li[1]*, Anna von Schantz[2], Madelen Fahlstedt[2], Peter Halldin[1,2]**
[1]Division of Neuronic Engineering, Department of Biomedical Engineering and Health Systems, KTH Royal Institute of Technology, Huddinge, Sweden
[2]Mips AB, Täby, Sweden
*Correspondence: xiaogai@kth.se

## S2 File: Skull stress and brain strain from Helmet-B

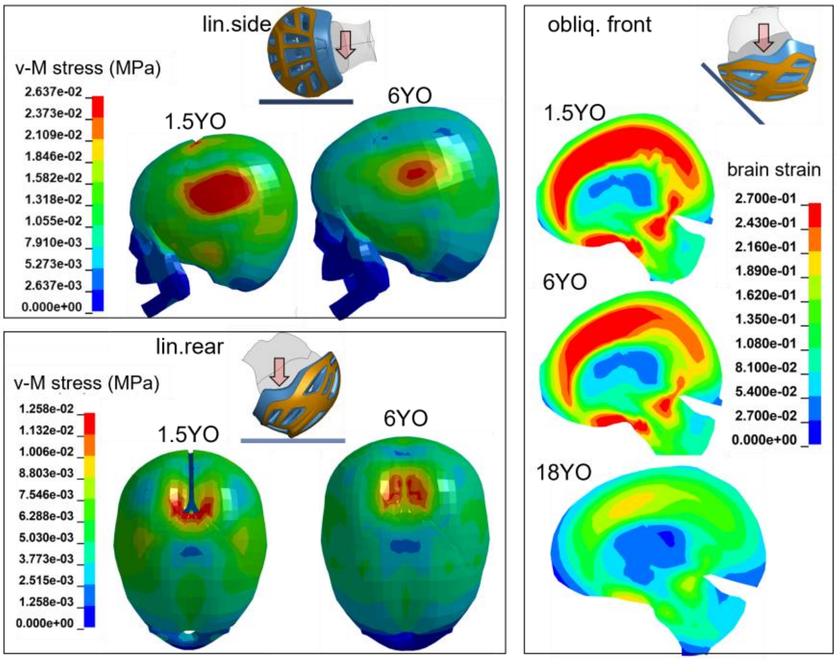


**Fig. A1** Age-dependence maximum v-M stress in the skull (a,b) and of brain strain (c) with *Helmet-B*. A sagittal plane of brain strain (1.5, 6, and 18YO oblique front) and skull stress (1.5 and 6YO linear side and linear rear) captured when peak value occurs (illustration with *Helmet-A*).
